# Treatment and outcome of patients with Graves’ disease and metastatic differentiated thyroid cancer

**DOI:** 10.2478/raon-2023-0034

**Published:** 2023-09-04

**Authors:** Nikola Besic, Barbara Vidergar-Kralj

**Affiliations:** Institute of Oncology Ljubljana, Ljubljana, Slovenia; Faculty of Medicine, University of Ljubljana, Ljubljana, Slovenia; Department of Nuclear Medicine, Institute of Oncology Ljubljana, Ljubljana, Slovenia

**Keywords:** differentiated thyroid cancer, metastases, Graves’ disease, treatment

## Abstract

**Background:**

The aim of the study was to report on the experience in a single tertiary cancer center about the treatment and outcome of patients with Graves’ disease (GD) and metastatic thyroid cancer as compared with patients without GD in our country.

**Patients and methods:**

Altogether, 28 patients (8 males, 20 females; 49–85 years of age; median 74 years) were treated because of differentiated thyroid cancer and distant metastasis at the time of diagnosis during a 10-year period (from 2010 to 2019) in the Republic of Slovenia. The subject of our retrospective study were four patients (three men, one female; 64–76 years of age, median 73 years) who had Graves’ disease and metastatic thyroid cancer.

**Results:**

The mean age of patients without GD and with GD was 74 years and 71 years, respectively (p = 0.36). There was a trend for male predominance in patients with GD (p = 0.06). There was no statistical difference in size of primary tumors, pT stage or pN stage between the group of patients without GD and with GD. The median length of follow-up was 3.33 years (range 0.04–7.83) and 5-year disease-specific survival was 51%. One of four patients with GD and 14 of 24 patients without GD died of thyroid cancer. There was no statistical difference in disease-specific survival between patients’ group of without GD and with GD (p = 0.59).

**Conclusions:**

In our country Slovenia, 14% of patients with metastatic differentiated thyroid carcinoma at the time of diagnosis had Graves’ disease. There was no difference in the treatment, outcome or survival of patients with GD in comparison to those without GD.

## Introduction

Graves’ disease (GD) is a systemic autoimmune disease directly caused by circulating antibodies against TSH receptor (anti-TSH-R) that bind to the thyrotropin receptor (TSH-R), subsequently inducing the production and release of thyroid hormone, proliferation of thyrocytes, and enlargement of the thyroid gland.^1,2^ High serum anti-TSH-R antibodies were reported to stimulate the growth of thyroid cancer and metastasis.^3^ A recent meta-analysis demonstrated an increased risk of distant metastasis at the time of cancer diagnosis in patients with differentiated thyroid cancer and GD in comparison to those without GD.^4^

There are only limited data in the literature about treatment of patients who have Graves’ disease and metastatic thyroid cancer, probably because the prevalence of GD in patients with thyroid cancer is very low, and the present guidelines do not give specific recommendations for the treatment of patients with differentiated thyroid cancer who have GD.^5,6^ However, Pellegriti *et al*.^7^ reported increased disease-specific mortality in patients with GD in comparison with matched euthyroid patients with thyroid cancer. Recently, we reported on a case report of a patient with simultaneous hormone-active metastatic Hürthle cell thyroid cancer and Graves’ disease which was treated by a combined multimodal treatment, but despite treatment, the disease rapidly progressed and the patient died due to distant metastases 28 months from diagnosis.^8^ The aim of the study was to report on experience in a single tertiary cancer center about the treatment and outcome of patients with Graves’ disease and metastatic thyroid cancer in comparison to those without Graves’ disease and metastatic thyroid cancer in our country Slovenia.

## Patients and methods

### Study population

There were 1524 patients (354 males and 1170 females, 11–91 years of age, median 51 years) with a differentiated thyroid carcinoma registered by The Cancer Registry of Republic of Slovenia during a 10-year period (from 2010 to 2019). During this period, altogether 28 patients (eight males, 20 females; 49–85 years of age; median 74 years) were treated because of differentiated thyroid cancer and distant metastasis at the time of diagnosis at our Institute. The subject of our retrospective study were four patients (three men, one female; 64–76 years of age, median 73 years) who had Graves’ disease and metastatic thyroid cancer. The Protocol Review Board and Ethics Committee of the Institute of Oncology on 9^th^ December 2020 (ERID-KSOPKR-0082/2020, ERIDEK-0083/2020, ERIDNPVO-0040/2020) reviewed and approved the study, which was conducted in accordance with the ethical standards prescribed in the Declaration of Helsinki. For retrospective studies, informed consent is not necessary according to the national regulations. The need for consent was waived by the Institutional Review Board and Ethics Committee of the Institute of Oncology Ljubljana.

Thyrotoxicosis with concomitant thyroid cancer was detected in 5 of 28 patients with distant metastases. Anti-TSH-R antibodies were not measured in all our patients. However, anti-TSH-R antibodies were measured in all patients with hyperfunctioning primary tumors or hyperfunctioning metastases. Elevated anti-TSH-R antibodies were detected in 4 of our 28 patients with distant metastases. All four patients with GD had a hyperfunctioning primary tumor as well as hyperfunctioning metastases.

A chart review for each patient with distant metastases was performed. All histological slides of our patients with metastatic differentiated thyroid cancer were examined by the pathologist experienced in thyroid pathomorphology. Distant metastases were diagnosed by clinical examination and additional diagnostic procedures, including lung and/or bone X-ray, ultrasonography, ultrasound guided fine-needle aspiration biopsy, radionuclide investigations with radioiodine and ^18^F-FDG PET/CT, computed tomography, and/or nuclear magnetic resonance imaging. The tumor stage, presence of regional and/or distant metastases, as well as residual tumor after surgery were assessed by the 8^th^ edition of TNM clinical classification according to the UICC criteria from 2017.^9^ Data on patients’ age, gender, disease history, presence of Graves’ disease, extent of cancer, histomorphological characteristics, mode of cancer specific therapy, outcome, and survival were collected. The clinical and pathological characteristics of the tumors are presented in Table 1. The treatment of patients and their outcome are presented in Table 2.

**TABLE 1.** Clinical characteristics and pathological characteristics of tumors

**Factor**	**Subgroup**	**All patients (N = 28)**	**Without Graves’ disease (N = 24)**	**With Graves’ disease (N = 4)**	**p-value**
**Mean age of patients (year)**		73.86	74.25	71.50	0.359
**Mean primary tumor size (cm)**		5.604	5.483	6.325	0.355
**Gender**	Female	20	19	1	0.058
	Male	8	5	3	
**Age (years)**	54 or less	1	1	0	1.00
	55 or more	27	23	4	
**Hyperthyreosis at presentation**	No	23	23	0	0.001
	Yes	5	1	4	
**Functional metastases**	No	26	24	2	0.016
	Yes	2	0	2	
**Tumor diameter (cm)**	0–4	9	8	1	1.00
	4.01 and more	19	16	3	
**pT tumor stage**	pTx, pT1 or pT2	7	6	1	0.522
	pT3	7	5	2	
	pT4	14	13	1	
**N stage**	N0	20	17	3	1.00
	N1 or N2	8	7	1	
**M stage**	M0	0	0	0	-
	M1	28	24	4	
**Type of metastases**	Lung only	11	10	1	0.759
	Bones and others	12	9	3	
	Lungs and others without bones	5	5	0	
**Single organ metastases**	No	14	11	3	0.596
	Yes	14	13	1	
**Tumor type**	Papillary	14	12	1	0.279
	Hürthle	6	4	2	
	Follicular	5	5	0	
	Poorly differentiated	3	3	1	
**Tumor differentiation (N = 19)**	Well	6	5	1	1.00
	Moderate or poor	13	12	1	

**TABLE 2.** Treatment of patients and their outcome

**Factor**	**Subgroup**	**All patients (N = 28)**	**Without Graves’ disease (N = 24)**	**With Graves’ disease (N = 4)**	**p-value**
**Thyroid surgery**	No	8	7	1	1.00
	Yes	20	17	3	
**Thyroid surgical procedure**	Total or near-total thyroidectomy Lobectomy or less	18	15	3	1.00
		10	9	1	
**Residual tumor after surgery**	No surgery	8	7	1	0.916
	R0 (without residual tumor)	11	9	2	
	R1	2	2	0	
	R2	7	6	1	
**Neck dissection**	No	24	21	3	0.481
	Yes	4	3	1	
**Surgery of distant metastases**	No	26	24	2	0.016
	Yes	2	0	2	
**RAI ablation after surgery**	No	10	9	1	1.00
	Yes	18	15	3	
**Therapy with RAI**	No	10	9	1	1.00
	Yes	18	15	3	
**EBRT to the neck**	No	21	17	4	0.545
	Yes	7	7	0	
**EBRT (any site)**	No	15	13	2	1.00
	Yes	13	11	2	
**Preoperative chemotherapy**	No	25	23	2	0.045
	Yes	3	1	2	
**Chemotherapy**	No	22	20	2	0.191
	Yes	6	4	2	
**Targeted therapy**	No	18	16	2	0.601
	Yes	10	8	2	
**Outcome**	Alive with disease	9	7	2	0.461
	Dead of disease	15	14	1	
	Dead of other causes	4	3	1	

RAI = radioiodine

The majority of patients received multimodal treatment. Surgery is the mainstay of the treatment of primary tumors in differentiated thyroid cancer. All surgically treated patients had primary surgery and all other cancer-specific therapies (surgery, radioiodine (RAI) ablation of thyroid remnant, RAI therapy, external beam radiotherapy (EBRT) and/or systemic therapy) at the Institute of Oncology. All patients received therapy with L-thyroxine for TSH suppression.

### Follow-up

All patients had a follow-up exam at our Institute at least twice per year. This consisted of obtaining medical history, a physical examination, and determining serum Tg concentration. Imaging with radioiodine scintigraphy and/or ^18^F-FDG PET/CT, computed tomography, and/or nuclear magnetic resonance was conducted once a year. It was also conducted whenever Tg concentration increased or clinical symptoms suggested that the disease had progressed.

### Survival

Cause-specific and overall survival was defined as the period from primary cancer treatment to death or the last follow-up. The median duration of follow-up was 3.33 years (range 0.04–7.83 years).

### Statistical analysis

The Student t-test or the Mann–Whitney U-test was used according to data distribution. The association between categorical variables was tested by the chi-square test or Fisher's exact test, as appropriate. All comparisons were two-sided and a p-value <0.05 was considered statistically significant. The survival curves were calculated according to the Kaplan-Meier method. A multivariate statistical analysis was not performed because of the small number of patients. The statistical package PASW 18 (SPSS Inc., Chicago, IL, USA) was used for the analysis.

## Results

### Patients with Graves’ disease

A 71-year-old patient with GD was presented to a surgical oncologist because of a 2.3 cm primary papillary thyroid cancer with lung metastases. He had hyperthyroidism and GD after iodine exposure, heart failure, generalized arteriosclerosis, gangrene of the lower extremity, and type 2 diabetes. He had just had a myocardial infarction with stented coronary arteries. Due to the poor general condition and accompanying diseases, we did not decide on surgical intervention or oncological treatment. He died after two months due to deterioration of heart function.

A total thyroidectomy was performed on a 76-year-old patient with hyperthyroidism due to GD and cytological suspicion of papillary thyroid cancer. Before the operation, she received thyrostatic drugs. Histologically, it was a poorly differentiated thyroid carcinoma, 6.5 cm in diameter with extensive capsular invasion, but no vascular invasion or extrathyroidal spread. The metastases were not functionally active. After hormonal withdrawal she received 150 mCi of RAI, which accumulated in the lungs, skeleton, left kidney and right paracolic area. After six months and another six months, she received 152 mCi and 145 mCi of RAI after hormonal withdrawal, which accumulated in the same places. After nine months, she received the 4^th^ and last 152 mCi of radioiodine and accumulation was less intense. After another nine months, there was no more accumulation in the metastases on RAI whole body scan, and ^18^F-FDG PET-CT showed the progression in the 5^th^ left rib and right iliac bone. The patient was still asymptomatic. The skeletal lesions with a progression of disease were treated with EBRT. Anti-TSH-R levels were elevated all the time. The Tg value fell from 11982 ng/mL before surgery to 9598 ng/mL before the first RAI therapy, and then slowly declined. Titres of anti-Tg antibodies were not increased. At the time of progression 34 months after the first treatment, Tg value was only 280 ng/mL, anti-Tg antibodies were not increased, but TPO antibodies were increasing. Because the patient was asymptomatic, the medical oncologist has not yet decided on systemic treatment and the patient has been on active surveillance for seven months since progression of the disease was diagnosed.

**FIGURE 1.**
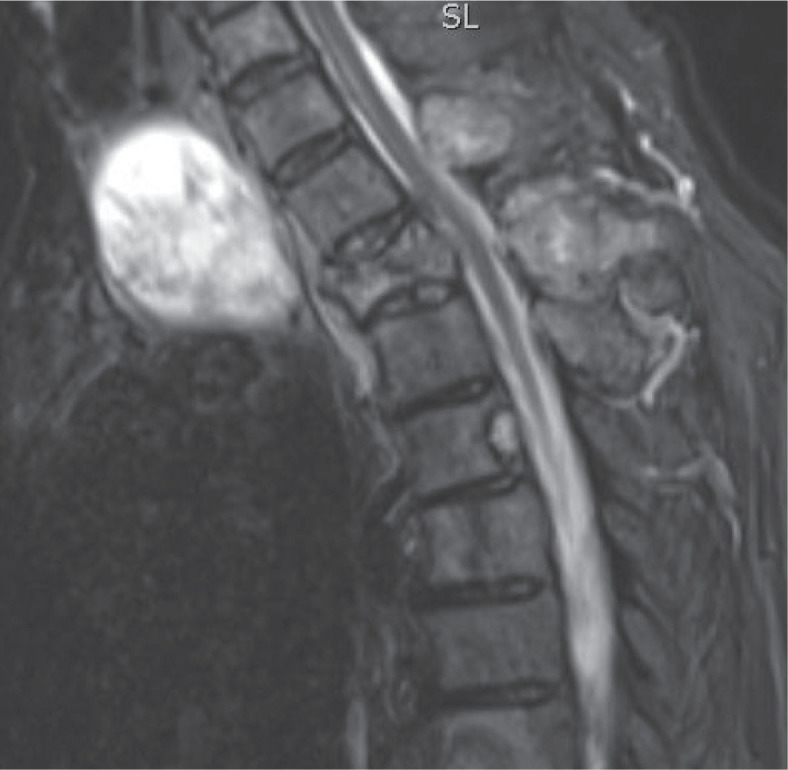
A 75-year-old patient was operated on after preparation with thiamazole for a pathological fracture of Th2. Spinal stabilization was performed.

A 75-year-old patient was operated on after preparation with thiamazole for a pathological fracture of Th2 (Figure 1) and spinal stabilization was performed in October 2017. Postoperatively, he was irradiated in the area of the thoracic spine with sensitization with weekly low doses of vinblastine and doxorubicin. In December 2017, a total thyroidectomy was performed. Histological examination showed a 7 × 5.5 cm oncocytic tumor with regressive changes after chemotherapy. In January 2018, T3 hyperthyroidism occurred. In February 2018, the patient received 151 mCi of RAI, which accumulated in many places in the spine. In March 2018, the RAI non-avid tumor in the sternum and in the right iliac bone was treated with EBRT. Because of side effects of bisphosphonates therapy, the patient discontinued this therapy. In August 2018, he had T3 hyperthyroidism again. After preparation with thiamazole, he received 160 mCi of RAI in October 2018. It accumulated in the skeletal metastases. Due to poor accumulation of RAI in Th5, he was referred to a medical oncologist, who did not decide on systemic therapy. In March 2019, he received 161 mCi RAI, the accumulation of RAI in metastases was less intense. In February 2020, he was asymptomatic, but ^18^F-FDG PET-CT showed a progression of metastases, so therapy with 166 mCi of RAI was administered. Only some metastases accumulated RAI. In April 2020, MRI showed progression of metastasis in Th5 and again T3 hyperthyroidism was diagnosed, therefore therapy with sorafenib (200 mg + 400 mg) was initiated. Because of the side effects, the dose was reduced to half, which he tolerated better. But after four months, the patient stopped this therapy due to side effects. He was asymptomatic until April 2022. At that time ^18^F-FDG PET-CT showed new metastases in the skeleton and T3 hyperthyroidism recurred. Therapy with lenvatinib was initiated on May 2022. For the first time since the beginning of cancer treatment, anti-TSH-R levels fell to the normal level three months after initiation of treatment with lenvatinib. The level of Tg also dropped from 5838 ng/mL in May 2020 to 2360 ng/mL in August 2022.

A 64-year-old male patient had clinical signs of hyperthyroidism and a tumor measuring 9 cm in diameter of the left thyroid lobe, metastatic neck lymph node and metastases in the lungs, mediastinum, and in the 8^th^ right rib measuring 20 × 5.6 × 4.5 cm, in the left acetabulum measuring 9 × 9 × 3 cm and in the skull measuring 5 × 4 × 2 cm.^8^ The region of the left hip had been irradiated with concomitant doxorubicin 20 mg once weekly. When hyperthyroidism was controlled with thiamazole, a total thyroidectomy was performed. Persistent T3 hyperthyroidism, most likely caused by anti-TSH-R stimulated T3 production in a large metastasis in the 8^th^ right rib, was cured by rib resection. The patient was treated with three RAI therapies. But after a short interval, the disease progressed despite treatment with RAI and therapy with sorafenib. The patient died due to distant metastases 28 months from the beginning of treatment.

### Histology, age of patients and type of metastases

At presentation, 5 of 28 patients had hyperthyroidism and four of them had GD. Two patients with GD had functional metastases.

Papillary carcinoma, Hürthle cell carcinoma, follicular and poorly differentiated thyroid carcinoma were diagnosed in 13, 6, 5 and 4 patients, respectively. Graves’ disease was present in two patients with Hürthle cell carcinoma, one with papillary and one with poorly differentiated thyroid carcinoma (p = 0.28).

The mean age of patients without GD and with GD was 74 years and 71 years, respectively. Age of patients with and without GD was not statistically different (p = 0.36). There was a trend for male predominance in patients with GD (p = 0.06). Mean primary tumor size in patients without GD and with GD was 5.5 and 6.3 cm, respectively (p = 0.36). There was no statistical difference in pT stage (p = 0.52) or pN stage (p = 1.00) between the group of patients without GD and with GD.

Initial sites of metastases were: lungs in 24 cases, bones in 12 cases, mediastinum in eight cases, liver in two cases and skin in one case. Single and multiple organ metastases were present in 14 and 14 patients, respectively. Lung metastases only, bone metastases only and skin metastases only were present in eleven patients, two patients and one patient, respectively. There was no statistical difference in distribution of metastases between patients without GD and with GD.

### Treatment

Palliative treatment was only applied in four cases: in one due to severe comorbidities, and in three due to very advanced cancer. The data on the type of surgery for primary tumors and treatment of distant metastasis are listed in Table 2. Total or near-total thyroidectomy is considered a proper surgical procedure for thyroid cancer; however, it was performed in only 18 of 28 patients. It was not performed because of inoperable tumors (N = 8), very advanced age of the patient, severe comorbidities (N = 1), or if the patient refused surgical procedure (N = 1). Initial treatment in three patients with a locally advanced tumor was neoadjuvant chemotherapy. Tumor size decreased in all patients: by more than 30% in two patients, and by less than 30% in one patient. Metastases in regional lymph nodes were surgically treated as a part of primary surgical procedure by functional radical neck dissection in four patients. Surgical treatment of distant metastases was more common in patients with GD in comparison to those without GD (p = 0.016). Surgical therapy of bone metastases was conducted on two patients: a resection of the 8^th^ right rib with hormone active metastases in one patient and a resection of metastasis in the 2^nd^ thoracic vertebra due to pathological fracture and narrowing of the spinal canal in another patient.

RAI was used for the ablation of thyroid remnant tissue in 18 (64%) patients. The ablation dose was 3.4–4.8 GBq (92–129 mCi) of RAI. RAI was used also for treatment of distant metastases with empiric dose of 3.7–7.4 GBq (100–200 mCi) in 14 patients. These 18 patients received altogether 31 therapies with RAI (range 1–4; median 2) and a dose 11.47–24.72 GBq (310–668 mCi; median 535 mCi).

Altogether, six patients were treated with chemotherapy, while kinase inhibitors were used in ten patients. EBRT was done in a total of 13 patients, of whom seven received EBRT to the neck and superior mediastinum.

### Survival

**FIGURE 2.**
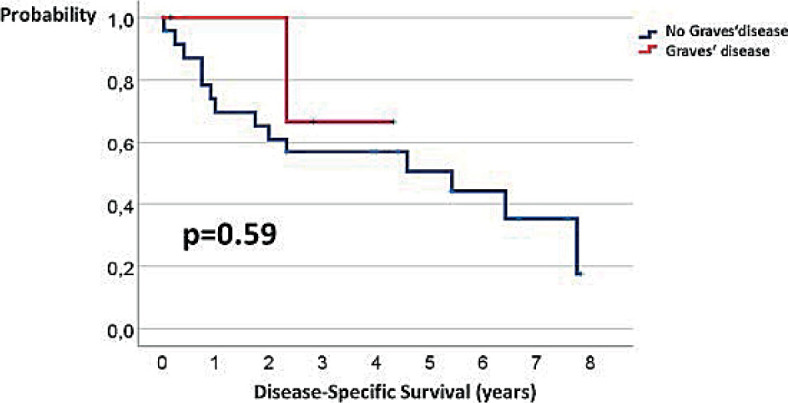
Disease-specific survival of patients with Graves’ disease (GD) and without GD.

**FIGURE 3.**
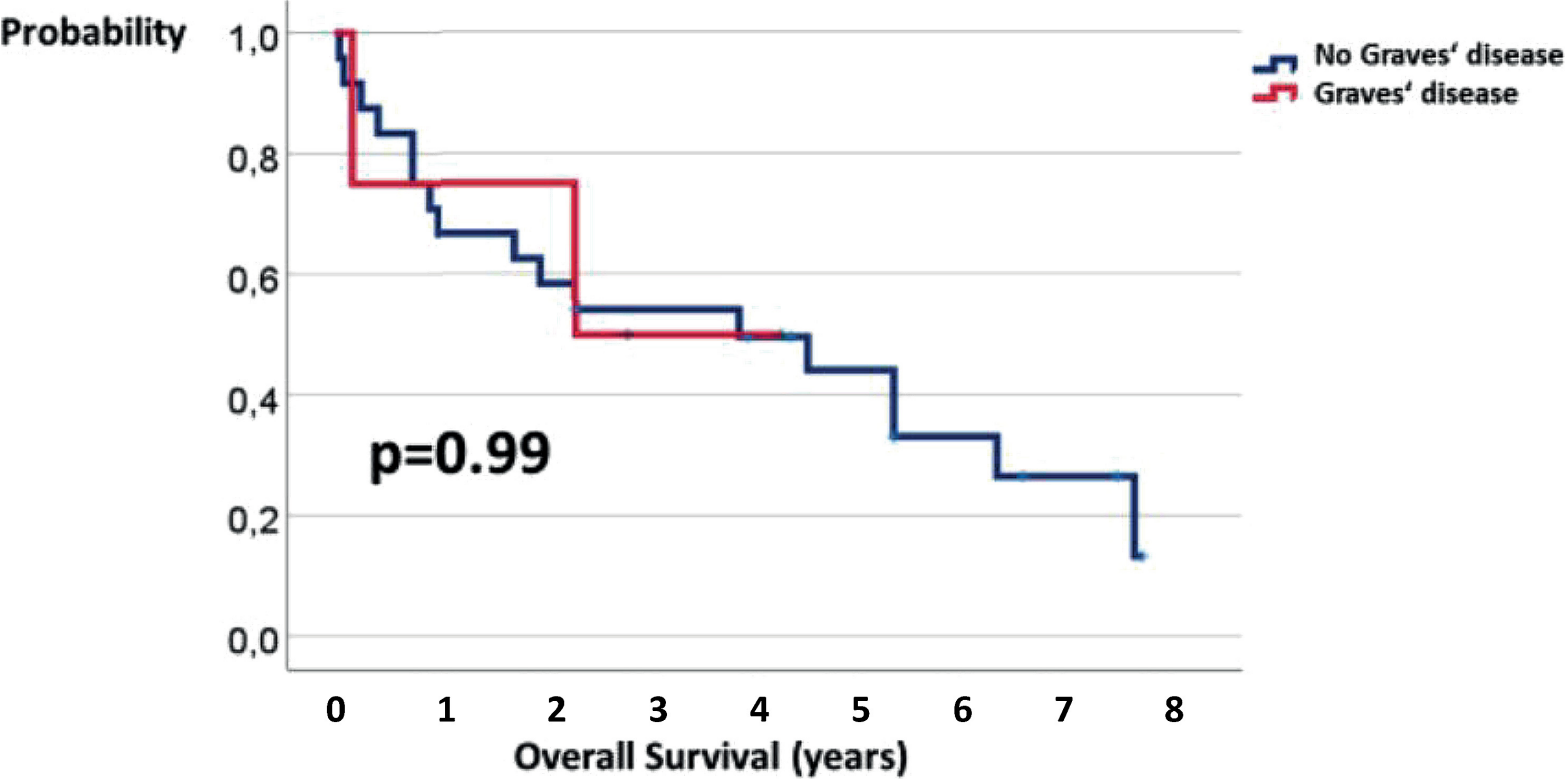
Overall survival of patients with Graves’ disease (GD) and without GD.

Patients were followed for 0.04–7.83 (median 3.33) years. Disease-specific survival of our 28 patients ranged from 0.04 to 7.83 years. The 5-year disease-specific and overall survival was 51% and 44%, respectively. The 3-year disease-specific survival of patients with and without Graves’ disease were 67% and 57%, respectively (Figure 2). The length of disease-specific survival in patients with and without Graves’ disease was not statistically different (p = 0.59). The 3-year overall survival of patients with and without Graves’ disease were 50% and 54%, respectively (Figure 3). The length of overall survival in patients with and without Graves’ disease was not statistically different (p=0.99).

In 18 patients who had total thyroidectomy and RAI therapy, the 5-year disease-specific and overall survival was 70% and 61%, respectively. The 3-year disease-specific survival of patients with and without Graves’ disease were 66% and 81%, respectively. The length of disease-specific survival in patients with and without Graves’ disease was not statistically different (p = 0.66). In these 18 patients, the 3-year overall survival of patients with and without Graves’ disease were 66% and 76%, respectively. The length of overall survival in patients with and without Graves’ disease was not statistically different (p = 0.91).

By the end of the study, nine patients were still alive (2.3–7.83 years, median 4.42 years), four patients died of causes unrelated to primary disease, while 15 patients died of thyroid carcinoma. Of the latter, eleven patients died of distant metastases, one of uncontrolled locoregional disease, and the remaining three patients died of distant and locoregional progression of the disease. Two of four patients with Graves’ disease are alive, at the time of writing for 34 and 52 months. Both have slow progression of RAI non-avid metastases; one is on systemic multikinase inhibitor therapy, while the other is still asymptomatic and on active surveillance before initiation of systemic therapy.

## Discussion

The aim of our study was to report on our experience about the treatment and outcome of patients with Graves’ disease and metastatic thyroid cancer in comparison to those without Graves’ disease and metastatic thyroid cancer in our country. In our country, 14% of patients with metastatic differentiated thyroid carcinoma at the time of diagnosis had Graves’ disease. There were no significant differences in the oncological treatment of patients with GD in comparison to those without GD. Cancer-specific and overall survival of patients with GD was not significantly shorter in comparison to those without GD.

Functioning metastatic thyroid carcinoma is a rare disease.^10^ Qiu *et al*.^10^ reported that the prevalence of hyperfunctioning metastases in patients with follicular thyroid carcinoma was 5/38 (13%). However, hyperfunctioning metastatic thyroid carcinoma with concomitant Graves’ disease is an even rarer condition. Our data show that it was present in 7% of patients with distant metastases at the time of diagnosis.

Treatment of patients with hyperfunctioning metastases of thyroid cancer and GD is a challenging task. For the treatment of patients with hyperfunctioning thyroid carcinoma, there are two aims: to control hyperthyroidism, as well as the cancer.^11^ Unfortunately, the current management guidelines for differentiated thyroid cancer does not recommend any specific management for patients with metastatic cancer and GD.^4,5^

Liu *et al*.^11^ reported that total thyroidectomy may be the optimal primary treatment option for patients with functional primary tumor and metastases which are not hyperfunctioning. In such cases a total thyroidectomy reduces the dose of RAI required to treat the metastatic lesions. But in patients with hyperfunctioning metastatic lesions with non-functioning primary thyroid carcinoma, a total thyroidectomy may lead to deterioration of hyperthyroidism, as the majority of hormones are produced by metastatic lesions.^11^ Such was the case in one of two of our patients with hyperfunctioning metastases. In a systematic review of the literature, Liu *et al*.^11^ reported that, after total or subtotal thyroidectomy, a transient improvement of hyperthyroidism was obtained in only one of five patients, while in four patients, hyperthyroidism persisted. Furthermore, one of four patients succumbed to thyroid crisis 12 days after surgery. Likewise, Girelli *et al*.^12^ reported on a case of extremely severe hyperthyroidism due to pelvic bone metastasis in which hyperthyroidism worsened after total thyroidectomy and after the first dose of RAI. But in their case, the administration of methimazole, prednisone and multiple, fractioned and small doses of radioiodine cured the hyperthyroidism and stabilized the neoplastic growth.^12^ Our experience is similar to the report by Liu *et al*.^11^, because after total thyroidectomy, hyperthyroidism persisted in both our patients with hyperfunctioning metastases. In one of them, therapy with thiamazole, RAI therapy, a combination of EBRT and concomitant doxorubicin chemotherapy cured hyperthyroidism caused by functional metastasis in bone metastasis in the sternum and pelvis. In the other patient, on the other hand, a surgical resection of the 8^th^ right rib in which functional metastasis caused a T3 hyperthyroidism was needed in order to cure hyperthyroidism.^8^ Our second case confirm the opinion, that in cases with a metastatic lesion which is resistant to RAI and the functioning lesion is resectable, surgery is a good treatment option.^11^

RAI is an essential part of the treatment of hyperfunctioning metastatic lesions.^10^ But therapy with RAI in patients with functioning metastases may result in thyroid storm and death.^11,13^ To avoid a possible thyroid storm, antithyroid medication is required before treatment with RAI.^11^ In the literature review, Fu *et al*.^14^ reported that activity of RAI to treat hyperfunctioning metastases varied from 13 mCi to 200 mCi.^14,15,16,17^ Severe hyperthyroidism can be improved with repeated low-dose radioiodine therapy.^16^ High doses of RAI could cause a large amount of tumor cell destruction, releasing a burst of thyroid hormones and causing thyrotoxic storm if the patients are not adequately prepared before and treated after RAI.^14^ Glucocorticoids and antithyroid medications should be used prior to surgery and RAI treatment to avoid the occurrence of thyrotoxic storm, as well as during RAI treatment in order to inhibit thyroid hormone synthesis and peripheral conversion of T4 to T3.^13^

Another effective evolving treatment modality for progressive metastatic thyroid cancer is systemic therapy.^8,18^ Systemic therapy with a multikinase inhibitor, sorafenib, was effective in two of our patients. The effect before cancer progression lasted eight and 24 months. Furthermore, hyperthyroidism was also prevented after therapy with sorafenib in both cases. In one of them, sorafenib was stopped due to side effects after four months. After progression of the disease and recurrence of hyperthyroidism, lenvatinib effectively prevented disease progression and cured hyperthyroidism. Also, Danilovic *et al*.^18^ described that targeted therapy with lenvatinib is an option for the control of hyperfunctioning metastases.

External beam radiotherapy is the treatment of choice for inoperable distant metastases and/or large functional metastases. Unfortunately, it does not always prevent hyperthyroidism.^16^

Premoli *et al.*^19^ reported that there was no association between baseline anti-TSH-R levels and outcome in patients with differentiated thyroid carcinoma associated with GD. But, Valenta *et al.*^20^ reported that in one of three patients with metastatic follicular thyroid cancer-causing hyperthyroidism associated with elevated anti-TSH-R, level of anti-TSH-R declined after two RAI treatments with improvement in thyrotoxicosis. Also, Basaria *et al*.^21^ reported in a patient with functional metastases of papillary thyroid cancer and GD, a decline in the level of anti-TSH-R to 87% of normal range after three RAI therapies. The effect of RAI therapy was confirmed with CT investigation and with decrease of thyroglobulin level which on TSH suppression declined from 2280 ng/dL to 55 ng/dL.^21^ In three of our patients with GD, levels of anti-TSH-R have not declined after therapy with RAI or with sorafenib. However, three months after initiation of therapy with lenvatinib, the level of anti-TSH-R declined dramatically in the patient.

One of the aims of our study was to compare outcome of patients with and without GD. Median survival of our patients with differentiated thyroid cancer with GD was 31 months. The 3-year disease-specific survival of patients with and without GD were 67% and 57%, respectively. The length of disease-specific survival in patients with and without GD was not statistically different (p = 0.59). Similar survival was reported by Als *et al*.^22^ who treated five patients with functional metastatic differentiated carcinoma with median survival of 39 months. Our results are also in agreement with a systematic review and meta-analysis of 25 studies which included 987 patients with differentiated thyroid cancer with GD and 2,064 patients with differentiated thyroid cancer without GD, which showed no difference in cancer related mortality and recurrence/persistence during follow-up.^4^

Our study has several limitations. The first limitation is the retrospective nature. Another limitation is a low number of all cases with metastatic thyroid cancer. Furthermore, only two of four patients with GD had hyperfunctioning metastases. However, to our knowledge, there are no data in the literature about the national incidence of patients with metastatic thyroid cancer and concomitant GD. Furthermore, notification of cancer has been compulsory in Slovenia since the foundation of the Cancer Registry of Republic of Slovenia in 1950 and prescribed by law^23^, so our data about incidences represent reliable population-based data. Additionally, all patients with thyroid cancer are treated at the Institute of Oncology in Ljubljana, so our data represent a population-based incidence of GD among patients with metastatic differentiated thyroid cancer.

## Conclusions

In our country, 14% of patients with metastatic differentiated thyroid carcinoma at the time of diagnosis had GD. There was a trend for male predominance in patients with GD. Treatment of patients with metastatic differentiated thyroid carcinoma at the time of diagnosis who have GD is multidisciplinary and includes surgical therapy, RAI therapy, systemic therapy and/or EBRT. There were no significant differences in the oncological treatment of patients with GD in comparison to those without GD. Cancer-specific and overall survival of patients with GD was not significantly shorter in comparison to those without GD.
